# Precision oncology in AML: validation of the prognostic value of the knowledge bank approach and suggestions for improvement

**DOI:** 10.1186/s13045-021-01118-x

**Published:** 2021-07-06

**Authors:** Marius Bill, Krzysztof Mrózek, Brian Giacopelli, Jessica Kohlschmidt, Deedra Nicolet, Dimitrios Papaioannou, Ann-Kathrin Eisfeld, Jonathan E. Kolitz, Bayard L. Powell, Andrew J. Carroll, Richard M. Stone, Ramiro Garzon, John C. Byrd, Clara D. Bloomfield, Christopher C. Oakes

**Affiliations:** 1 The Ohio State University Comprehensive Cancer Center, 460 West 12th Avenue, Columbus, OH 43210-1228 USA; 2 The Ohio State Comprehensive Cancer Center, Clara D. Bloomfield Center for Leukemia Outcomes Research, The Ohio State University, Columbus, OH USA; 3 Alliance Statistics and Data Center, The Ohio State University Comprehensive, Cancer Center, Columbus, OH USA; 4 Division of Hematology, Department of Internal Medicine, The Ohio State University Comprehensive Cancer Center, 400 West 12th Avenue, Wiseman Hall, Suite 455, Columbus, OH 43210-1228 USA; 5 Zucker School of Medicine At Hofstra/Northwell, Northwell Health Cancer Institute, Lake Success, NY USA; 6 Wake Forest Baptist Comprehensive Cancer Center, Winston-Salem, NC USA; 7 University of Alabama At Birmingham, Birmingham, AL USA; 8 Department of Medical Oncology, Dana-Farber/Partners CancerCare, Boston, MA USA; 9 The Ohio State University Comprehensive Cancer Center, 444 Tzagournis Medical Research Facility, 420 West 12th Avenue, Columbus, OH 43210-1228 USA

**Keywords:** Acute myeloid leukemia, Knowledge bank, Next-generation sequencing, Gene mutations, Clinical outcome

## Abstract

Recently, a novel knowledge bank (KB) approach to predict outcomes of individual patients with acute myeloid leukemia (AML) was developed using unbiased machine learning. To validate its prognostic value, we analyzed 1612 adults with de novo AML treated on Cancer and Leukemia Group B front-line trials who had pretreatment clinical, cytogenetics, and mutation data on 81 leukemia/cancer-associated genes available. We used receiver operating characteristic (ROC) curves and the area under the curve (AUC) to evaluate the predictive values of the KB algorithm and other risk classifications. The KB algorithm predicted 3-year overall survival (OS) probability in the entire patient cohort (AUC_KB_ = 0.799), and both younger (< 60 years) (AUC_KB_ = 0.747) and older patients (AUC_KB_ = 0.770). The KB algorithm predicted non-remission death (AUC_KB_ = 0.860) well but was less accurate in predicting relapse death (AUC_KB_ = 0.695) and death in first complete remission (AUC_KB_ = 0.603). The KB algorithm’s 3-year OS predictive value was higher than that of the 2017 European LeukemiaNet (ELN) classification (AUC_2017ELN_ = 0.707, *p* < 0.001) and 2010 ELN classification (AUC_2010ELN_ = 0.721, *p* < 0.001) but did not differ significantly from that of the 17-gene stemness score (AUC_17-gene_ = 0.732, *p* = 0.10). Analysis of additional cytogenetic and molecular markers not included in the KB algorithm revealed that taking into account atypical complex karyotype, infrequent recurrent balanced chromosome rearrangements and mutational status of the *SAMHD1*, *AXL* and *NOTCH1* genes may improve the KB algorithm. We conclude that the KB algorithm has a high predictive value that is higher than those of the 2017 and 2010 ELN classifications. Inclusion of additional genetic features might refine the KB algorithm.

**Supplementary Information:**

The online version contains supplementary material available at 10.1186/s13045-021-01118-x.

## To the Editor,

Risk-stratification schemas based on cytogenetic data and mutational status of selected genes, such as the 2010 and 2017 ELN genetic-risk classifications [1, 2], are widely used to predict the AML patients’ outcomes and guide therapeutic decisions. To increase accuracy of outcome prediction for individual patients, Gerstung et al. [3] developed a novel knowledge bank (KB) algorithm, which combined data on pretreatment clinical, cytogenetic, and gene mutation characteristics, treatment received, and outcomes from 1540 German AML patients [3]. Testing of several machine learning models revealed that inclusive, multistage statistical models scored best in predicting OS and probabilities of non-remission death, relapse death, and death in CR1. Although a relatively small study [4] confirmed prognostic usefulness of KB approach, to our knowledge, it has not been hitherto validated in a large, independent patient cohort. Therefore, we applied the KB algorithm to 1612 adults with de novo AML and investigated whether additional cytogenetic and molecular alterations might improve its accuracy. No patient receiving an allogeneic stem-cell transplantation in CR1 was included in the analyses (Additional file 1).

**Fig. 1:**
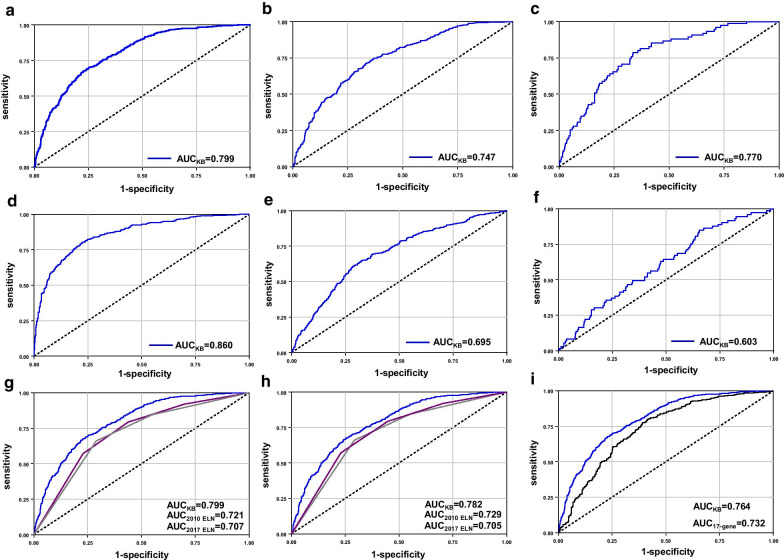
The receiver operating characteristic (ROC) curves illustrating the ability of the knowledge bank (KB) algorithm to predict 3-year overall survival rates in the **a** whole AML patient cohort, **b** younger adults with AML and **c** older adults with AML. The ROC curves illustrating the ability of the KB algorithm to predict additional outcome endpoints. **d** non-remission death, **e** relapse death and **f** death in first complete remission. The ROC curves illustrating the abilities of the KB algorithm (blue line), 2017 European LeukemiaNet (ELN) genetic-risk classification (gray line) and 2010 ELN genetic-risk classification (magenta line) to predict 3-year overall survival rates in the **g** whole cohort of patients with AML and **h** patients who did not die early. **i** The ROC curves showing the abilities of the KB algorithm (blue line) and the 17-gene stemness score (magenta line) to predict 3-year overall survival rates in 863 patients with RNA expression data available

We used ROC curves and the AUC to assess the ability of the KB approach to predict 3-year OS probability in comparison with the actual patient outcomes. The KB algorithm had a high AUC_KB_ = 0.799 (95% CI 0.777–0.821) for the entire patient cohort, for younger (< 60 years) patients AUC_KB_ = 0.747 (95% CI 0.717–0.776) and for older (≥ 60 years) patients AUC_KB_ = 0.770 (95% CI 0.716–0.824), for whom risk stratification is more difficult because they have generally poor prognosis (Fig. 1a–c).

Concerning other outcome endpoints, the KB algorithm was excellent for prediction of non-remission death (i.e., death within 3 years after diagnosis without CR1 achievement) with an AUC_KB_ = 0.860 (95% CI 0.838–0.882). For relapse death (i.e., death of patients achieving CR1 who relapsed and died within first 3 years), the predictive ability of the KB approach was worse (AUC_KB_ = 0.695, 95% CI 0.662–0.727). It was even worse for prediction of death in CR1, with a poor AUC_KB_ of 0.603 (95% CI 0.537–0.670; Fig. 1d–f).

Next, we compared the predictive values of the KB approach and of two well-established genetic-risk classifications, the 2010 [1, 5, 6] and 2017 ELN [2, 7, 8] classifications. Among all patients, the KB approach had the highest predictive value with AUC_KB_ = 0.799 (95% CI 0.777–0.821), followed by the 2010 ELN classification (AUC_2010ELN_ = 0.721, 95% CI 0.696–0.746) and the 2017 ELN classification (AUC_2017ELN_ = 0.707, 95% CI 0.682–0.732; Fig. 1g). Compared directly, the KB approach was significantly better than both the 2017 (*p* < 0.001) and 2010 (*p* < 0.001) ELN classifications.

When we performed the aforementioned comparisons after excluding early death patients, the KB approach still outperformed both the 2010 and 2017 ELN classifications, but the differences among classifications were smaller than in the entire patient cohort (Fig. 1h; Additional file 1).

We also compared the predictive value of the KB approach [3] with another AML risk classification, the 17-gene stemness score [9, 10], which is calculated as the weighted sum of the normalized expression values of 17 genes whose expression differs between leukemia stem cells and leukemic bulk blasts [9]. Among our 863 patients with RNA expression data available, the predictive values of the KB approach (AUC_KB_ = 0.764, 95% CI 0.733–0.800) and of the 17-gene stemness score (AUC_17-gene_ = 0.732, 95% CI 0.700–0.765) did not differ significantly (*p* = 0.10; Fig. 1i).

To determine whether genetic alterations not included in the KB algorithm might improve its performance, we compared the frequencies of 44 gene mutations and eight cytogenetic categories (listed in Additional file 1) between patients alive 3 years after diagnosis who were correctly predicted alive and patients falsely predicted to be dead. Three molecular and two cytogenetic markers were significantly different between the patient groups (Table 1).

**Table 1:** Predicted and observed frequencies of additional genetic markers in AML patients alive and those who were dead 3 years after diagnosis

Characteristic	Patients alive			Patients dead		
	Correctly predicted alive (%) *n* = 300	Falsely predicted dead (%) *n* = 222	p*	Falsely predicted alive (%) *n* = 368	Correctly predicted dead (%) *n* = 722	p*
*AXL*, mutated	1	2	< 0.001	3	0	0.09
*NOTCH1*, mutated	3	1	< 0.001	1	3	0.18
*SAMHD1*, mutated	1	2	0.04	1	0	0.04
Atypical complex karyotype, present	2	5	0.005	0	3	0.01
Recurrent but infrequent balanced rearrangements, present	1	2	0.04	0	2	0.17

*p-values for categorical variables are from Fisher’s exact test. *p*-values for continuous variables are from Wilcoxon rank sum test

To cross-validate these findings, we compared these markers’ frequencies between patients who died within first 3 years and were correctly predicted as dead and those falsely predicted to be alive. The frequencies of *SAMHD1* mutations and atypical complex karyotype (i.e., without 5q, 7q and 17p abnormalities) [11] were significantly different in both comparisons. Frequencies of *AXL* and *NOTCH1* mutations and of infrequent recurrent balanced chromosome rearrangements [12] were significantly different among patients alive and tended to be different among patients who died (Table 1).

Summarizing, we show that the KB algorithm has a high predictive value, higher than the 2017 and 2010 ELN classifications, and identify additional genetic factors that might improve it.

## Supplementary Information

**Additional file 1**. Supplementary Material.

## Data Availability

The datasets used and/or analyzed during the current study are available from the corresponding authors on reasonable request.

## Abbreviations

AML: Acute myeloid leukemia
OS: Overall survival
ELN: European LeukemiaNet
KB: Knowledge bank
CR1: First complete remission
CALGB: Cancer and Leukemia Group B
Alliance: Alliance for Clinical Trials in Oncology
ROC: Receiver operating characteristic
AUC: Area under the curve
