# Supplementary material for: Precision oncology in AML: validation of the prognostic value of the knowledge bank approach and suggestions for improvement
Source: J Hematol Oncol. 2021 Jul 6;14:107. doi: 10.1186/s13045-021-01118-x (PMC8261916; doi:10.1186/s13045-021-01118-x)

**Supplementary Information**

**Precision oncology in AML: validation of the prognostic value of the knowledge bank approach and suggestions for improvement**

Marius Bill,^1,^* Krzysztof Mrózek,^1,2^* Brian Giacopelli,^1,^* Jessica Kohlschmidt,^1,2,3^

Deedra Nicolet,^1,2,3^ Dimitrios Papaioannou,^1,4^ Ann-Kathrin Eisfeld,^1,2,4^

Jonathan E. Kolitz,^5^ Bayard L. Powell,^6^ Andrew J. Carroll,^7^ Richard M. Stone,^8^ Ramiro Garzon,^1,4^ John C. Byrd,^1,2,4^ Clara D. Bloomfield^1,4,†^

and Christopher C. Oakes^1,4,†^

^1^ The Ohio State University Comprehensive Cancer Center, Columbus, OH, USA

^2^ The Ohio State University Comprehensive Cancer Center, Clara D. Bloomfield

Center for Leukemia Outcomes Research, Columbus, OH, USA

^3^ Alliance Statistics and Data Center, The Ohio State University Comprehensive

Cancer Center, Columbus, OH, USA

^4^ Division of Hematology, Department of Internal Medicine, The Ohio State University,

Columbus, OH, USA

^5^  Northwell Health Cancer Institute, Zucker School of Medicine at Hofstra/Northwell,

Lake Success, NY, USA

^6^ Wake Forest Baptist Comprehensive Cancer Center, Winston-Salem, NC, USA

^7^ University of Alabama at Birmingham, Birmingham, AL, USA

^8^ Department of Medical Oncology, Dana-Farber/Partners CancerCare, Boston, MA,

USA

* Marius Bill, Krzysztof Mrózek and Brian Giacopelli contributed equally to this study.

^†^ Clara D. Bloomfield and Christopher C. Oakes contributed equally to this study as

senior authors.

**Participating institutions**

The following Cancer and Leukemia Group B (CALGB)/Alliance for Clinical Trials in Oncology (Alliance) institutions participated in this study and contributed at least five patients. For each of these institutions, the current or last principal investigator and the cytogeneticists who analyzed the cases are listed as follows:

The Ohio State University Medical Center, Columbus, OH: Claire F. Verschraegen, Karl S. Theil, Diane Minka and Nyla A. Heerema; North Shore University Hospital, Manhasset, NY: Jonathan E. Kolitz, Prasad R. K. Koduru, Ayala Aviram-Goldring and Chandrika Sreekantaiah; Wake Forest University School of Medicine, Winston-Salem, NC: Heidi D. Klepin, P. Nagesh Rao, Wendy L. Flejter and Mark Pettenati; Dana Farber Cancer Institute, Boston, MA: Harold J. Burstein, Ramana V. Tantravahi, Cynthia C. Morton and Paola Dal Cin; Roswell Park Cancer Institute, Buffalo, NY: Ellis G. Levine and AnneMarie W. Block; Washington University School of Medicine, St. Louis, MO: Nancy L. Bartlett, Michael S. Watson, Eric C. Crawford, Jaime Garcia-Heras, Peining Li and Shashikant Kulkarni; University of Chicago Medical Center, Chicago, IL: Hedy L. Kindler, Diane Roulston, Katrin M. Carlson, Yanming Zhang and Michelle M. LeBeau; University of North Carolina, Chapel Hill, NC: Matthew I. Milowsky and Kathleen W. Rao; University of Maryland Greenebaum Cancer Center, Baltimore, MD: Heather D. Mannuel, Joseph R. Testa, Maimon M. Cohen, Judith Stamberg and Yi Ning; University of Iowa Hospitals, Iowa City, IA: Umar Farooq and Shivanand R. Patil; Duke University Medical Center, Durham, NC: Jeffrey Crawford, Sandra H. Bigner, Mazin B. Qumsiyeh, John Eyre and Barbara K. Goodman; Dartmouth Medical School, Lebanon, NH: Konstantin H. Dragnev, Doris H. Wurster-Hill and Thuluvancheri K. Mohandas; Christiana Care Health Services, Inc., Newark, DE: Gregory A. Masters, Digamber S. Borgaonkar, Jeanne M. Meck, and Kathleen Richkind; Weill Medical College of Cornell University, New York, NY: Scott Tagawa, Ram S. Verma, Prasad R.K. Koduru, Andrew J. Carroll and Susan Mathew; Ft. Wayne Medical Oncology/Hematology, Ft. Wayne, IN: Sreenivasa Nattam and Patricia I. Bader; Western Pennsylvania Hospital, Pittsburgh, PA: Gene G. Finley and Gerard R. Diggans; Rhode Island Hospital, Providence, RI: Howard P. Safran, Teresita Padre-Mendoza, Hon Fong L. Mark, Shelly L. Kerman and Aurelia Meloni-Ehrig; SUNY Upstate Medical University, Syracuse, NY: Stephen L. Graziano, Larry Gordon and Constance K. Stein; University of Vermont Cancer Center, Burlington, VT: Peter A. Kaufman, Elizabeth F. Allen and Mary Tang; University of Alabama at Birmingham, Birmingham, AL: Lakshminarayanan Nandagopal and Andrew J. Carroll; University of Massachusetts Medical Center, Worcester, MA: William V. Walsh, Philip L. Townes, Vikram Jaswaney, Kathleen Richkind, Patricia Miron and Michael J. Mitchell; Eastern Maine Medical Center, Bangor, ME: Sarah J. Sinclair and Laurent J. Beauregard; Mount Sinai School of Medicine, New York, NY: Michael A. Schwartz and Vesna Najfeld; Long Island Jewish Medical Center, Lake Success, NY: Jonathan E. Kolitz, Alan L. Shanske, Prasad R. K. Koduru, Ayala Aviram-Goldring and Chandrika Sreekantaiah; University of California San Diego Moores Cancer Center, San Diego, CA: Lyudmila A. Bazhenova, E. Robert Wassman, Jr., Renée Bernstein and Marie L. Dell'Aquila; Walter Reed National Military Medical Center, Bethesda, MD: Karen G. Zeman, Rawatmal B. Surana, Digamber S. Borgaonkar, Karl S. Theil and Kathleen E. Richkind; Massachusetts General Hospital, Boston, MA: David Ryan, Leonard L. Atkins, Cynthia C. Morton and Paola Dal Cin; University of Tennessee Cancer Center, Memphis, TN: Harvey B. Niell and Sugandhi A. Tharapel; University of Illinois, Chicago, IL: John G. Quigley, Maureen M. McCorquodale, Kathleen E. Richkind and Valerie Lindgren; University of Missouri/Ellis Fischel Cancer Center, Columbia, MO: Puja Nistala, Jeffrey R. Sawyer, Tim Hui-Ming Huang and Linda M. Pasztor; University of Puerto Rico, San Juan, Puerto Rico: Eileen I. Pacheco, Leonard L. Atkins, Cynthia C. Morton and Paola Dal Cin; University of California at San Francisco, San Francisco, CA: Charalambos Andreadis and Kathleen E. Richkind; Virginia Commonwealth University, Richmond, VA: Zhijian Chen, Mary H. Hackney and Colleen Jackson-Cook; University of Minnesota, Minneapolis, MN: Robert A. Kratzke, Diane C. Arthur and Betsy A. Hirsch; University of Nebraska Medical Center, Omaha, NE: Apar Ganti and Warren G. Sanger; Georgetown University Medical Center, Washington, DC: Minnetta C. Liu and Jeanne M. Meck.

**Patients and treatment**

We investigated 1612 adult patients diagnosed with *de novo* AML (excluding acute promyelocytic leukemia) for whom pretreatment bone marrow (BM) or blood samples containing ≥20% leukemic blasts and outcome data were available. Patients with secondary or treatment-related AML were not included. No patient received an allogeneic hematopoietic stem-cell transplantation (allo-HSCT) in first complete remission (CR1) on study protocols, and off-study patients who received an allo-HSCT in CR1 were excluded from the analyses because their follow-up data are either incomplete or missing. The induction and consolidation regimes of each protocol are also highlighted in Supplementary Table S1. The patient cohort consisted of 1043 younger patients, aged <60 years (range, 17-59 years), and 569 older patients, aged ≥60 years (range, 60-92 years). One-hundred-seventy-three patients died within 30 days of treatment initiation (early death patients). The pretreatment characteristics of all analyzed patients and, separately, of patients younger than 60 years and those aged ≥60 years are shown in Supplementary Table S1, and their outcomes in Supplementary Table S2.

Almost all patients (97.6%) received similar cytarabine/daunorubicin-based treatment regimens on Cancer and Leukemia Group B (CALGB) trials [1-14]; 2.4% of patients were treated with decitabine+/-bortezomib [15]. The median follow-up for patients alive was 8.7 years. CALGB is now part of Alliance for Clinical Trials in Oncology. All patients were enrolled on companion protocols CALGB 8461 (cytogenetic studies), CALGB 9665 (leukemia tissue bank) and CALGB 20202 (molecular studies). Each patient provided written informed consent, and all study protocols were approved by the Institutional Review Boards at each participating center in accordance with the Declaration of Helsinki.

Patients were treated on the following CALGB/Alliance protocols: CALGB 8361 (n=2), 8525 (n=75), 8721 (n=2), 8821 (n=8), 8923 (n=27), 9022 (n=9), 9120 (n=2), 9222 (n=84), 9420 (n=22), 9621 (n=172), 9720 (n=233), 10201 (n=166), 10502 (n=38), 10503 (n=312), 10603 (n=71), 10801 (n=1), 11001 (n=10), 11002 (n=38), and 19808 (n=340). Treatment details are provided below and in the Supplementary Table 1.

Patients enrolled on CALGB 8525 were treated with induction chemotherapy consisting of cytarabine and daunorubicin, and were randomly assigned to consolidation with or without high-dose (i.e., 3 g/m^2^) cytarabine (HiDAC) followed by maintenance treatment [1]. The patient enrolled on CALGB 8721 was assigned to a regimen consisting of HiDAC plus asparaginase. The patient enrolled on 10801, received induction consisting of cytarabine in combination with daunorubicin. Patients enrolled on CALGB 8821 received intensive postremission therapy with cyclophosphamide/etoposide and diazaquone/mitoxantrone after induction consisting of cytarabine in combination with daunorubicin [2]. Patients enrolled on CALGB 9022 received induction chemotherapy consisting of cytarabine in combination with daunorubicin followed by consolidation with one cycle of HiDAC, a cycle of cyclophosphamide and etoposide, and one cycle of mitoxantrone and diaziquone [3]. Patients enrolled on CALGB 9120 received standard induction chemotherapy [4]. Patients enrolled on CALGB 9222 received induction chemotherapy consisting of cytarabine in combination with daunorubicin followed by consolidation with one cycle of HiDAC. Different doses of mitoxantrone were explored, and the consolidation treatment was randomized to three cycles of monotherapy with HiDAC or consolidation with one cycle of HiDAC, a cycle of cyclophosphamide and etoposide, and one cycle of mitoxantrone and diaziquone [5]. Patients enrolled on CALGB 9621 were treated similarly to those on CALGB 19808, as previously reported [6]. Patients on CALGB 10201 received induction chemotherapy consisting of cytarabine and daunorubicin, with or without the BCL2 antisense oblimersen sodium. The consolidation included two cycles of HiDAC (2 g/m^2^/d) with or without oblimersen [7]. For patients on CALGB 10502, bortezomib was added to both induction consisting of cytarabine and daunorubicin and to consolidation with two cycles of intermediate-dose cytarabine [8]. Patients enrolled on CALGB 10503 were assigned to receive induction chemotherapy consisting of cytarabine, daunorubicin, and etoposide. Upon achievement of CR, patients with core-binding factor AML (CBF-AML) received three courses of HiDAC. All other patients received HiDAC and etoposide for stem-cell mobilization followed by myeloablative treatment with busulfan and etoposide supported by autologous peripheral HSCT. After intensification, patients received the DNA methyltransferase inhibitor decitabine for maintenance [9]. Patients enrolled on CALGB 10603 were treated with cytarabine and daunorubicin followed by consolidation with HiDAC with or without PKC-412 [10]. Patients enrolled on CALGB 19808 were randomly assigned to receive induction chemotherapy with cytarabine, daunorubicin, and etoposide with or without PSC-833 (valspodar), a multidrug resistance protein inhibitor [11]. On achievement of CR, patients with CBF-AML were assigned to receive postremission therapy containing three courses of HiDAC. Patients with non-CBF AML were assigned to intensification with HiDAC and etoposide for stem-cell mobilization followed by myeloablative treatment with busulfan and etoposide supported by autologous peripheral blood HSCT. Patients on CALGB 8923 were treated with induction therapy consisting of cytarabine and daunorubicin and were randomly assigned to receive postremission therapy with cytarabine alone or in combination with mitoxantrone. Older patients enrolled onto CALGB 9420 or 9720 received induction chemotherapy consisting of cytarabine in combination with daunorubicin and etoposide, and were randomized to the arm with or without PSC-833 [12,13]. The PSC-833 arm was closed after random assignment of 120 patients because of a high number of early deaths. Patients on CALGB 9720 received a single cytarabine/daunorubicin consolidation course and were randomly assigned to low-dose recombinant interleukin-2 maintenance therapy or none. For patients treated on CALGB 11001, sorafenib was added to the induction and consolidation treatment consisting of daunorubicin and cytarabine and consolidation with HiDAC, followed by sorafenib maintenance [14]. Patients on CALGB 11002 received decitabine with or without addition of the proteasome inhibitor bortezomib, for both induction and postremission therapy [15].

**Cytogenetic and molecular analyses**

Cytogenetic analyses of pretreatment BM and/or blood samples were performed by the CALGB/Alliance-approved institutional laboratories using unstimulated short-term (24- or 48-h) cultures. All results were reviewed centrally by the Karyotype Review Committee [16].

Molecular analyses were performed as previously described [17,18]. Briefly, mononuclear BM or blood cells were enriched by Ficoll-Hypaque (GE Healthcare, Chicago, IL) gradient according to manufacturer’s guidelines and cryopreserved in liquid nitrogen until thawed at 37^o^C for analysis. DNA was extracted using the DNeasy Blood and Tissue Kit (QIAGEN, Hilden, Germany) following the manufacturer’s protocol. The mutational status of 80 protein-coding genes was determined centrally at The Ohio State University by targeted amplicon sequencing using the MiSeq platform (Illumina, San Diego, CA) [17]. As in the study of Gerstung et al. [19] all variants whose variant allele fractions were <0.05 were defined as not mutated. Additionally, variants were excluded if they occurred only in one read direction when sequenced in both directions, if the region contained many variants with low quality scores, or if they occurred in all analyzed samples including run controls. Samples with high background noise were excluded from analysis. Samples were considered non-evaluable and excluded for a specific gene if ≥85% of the amplicons covering the target regions within the coding sequence of the gene were sequenced to a depth of <15 reads.

The presence or absence of *FLT3* internal tandem duplications (*FLT3*-ITD) and *CEBPA* mutations were determined as described previously [20,21]. Overall, we assessed mutational status of 81 genes.

All patients were classified according to the 2017 ELN [22] and 2010 ELN [23] guidelines. For 863 patients with material available, we also derived a prognostic 17-gene stemness score as previously described [24].

**Statistical analyses**

Basic clinical characteristics were compared using the Fisher’s exact and Wilcoxon rank-sum tests for categorical and continuous variables, respectively [25]. For all analyses, we used the published KB algorithm using the available R code (<https://cancer.sanger.ac.uk/aml-multistage/>) [19]. We used receiver operating characteristic (ROC) curves as graphical plots to show the predictive value of the analyzed tests. The areas under the curve (AUC) are provided together with the 95% confidence intervals. An AUC=0.50 denotes lack of prediction ability, equal to that of random chance or a coin flip, whereas an AUC=1.00 (highest possible value) indicates perfect prediction ability. Commonly accepted criteria are that an AUC of 0.6-0.69, 0.7-0.79, 0.8-0.89, and ≥0.9 indicate, respectively, poor, fair, good and very good prediction ability [26,27]. However, since AML is a very heterogeneous disease, an AUC≥0.8 indicates a very strong prediction ability [26,27]. In our model with a binary outcome, that is, dead or alive at a defined time point, the AUC is equal to the more commonly known c-statistic [25-28]. To establish if patients were dead or alive after three years based on the probabilities generated by the KB algorithm, we used inverse transform sampling. All analyses were performed by the Alliance Statistics and Data Center on a database locked on October 1, 2019 using SAS 9.4 and TIBCO Spotfire S+8.2.

**Definition of clinical endpoints**

Clinical endpoints were defined according to generally accepted criteria [22]. A CR was defined as recovery of morphologically normal bone marrow and blood counts (i.e., neutrophil count ≥1.5 x 10^9^/l, with the exception for protocols CALGB 10503 and 10603, which required an absolute neutrophil count of ≥1.0 x 10^9^/l, and platelet count >100 x 10^9^/l), and no circulating leukemic blasts or evidence of extramedullary leukemia, all of which had to persist for ≥4 weeks. Overall survival was measured from the date of diagnosis to the date of death from any cause; patients not known to have died at last follow-up are censored on the date they were last known to be alive.

**The KB algorithm, missing data, a choice of a time point to predict OS of AML patients**

The KB algorithm [19] takes into account two demographic items, nine clinical parameters, 26 cytogenetic abnormalities, and mutational status of 58 genes (Supplementary Table S3). Notably, missing data are generally tolerated by the algorithm, and outcome predictions for individual patients are still possible if some of the aforementioned data are missing. Although we included as many parameters as possible for each patient to obtain the most accurate results, there were some missing data. Of the 58 gene mutations included in the KB algorithm [17], 44 were covered by our sequencing panel [29] and molecular analyses [20,21]. Importantly, we have analyzed all 29 gene mutations with the highest predictive value in the Gerstung et al. [19] study (Supplementary Table S3). All demographics and cytogenetics data were available for the whole cohort, and, among clinical parameters, only the lactic acid dehydrogenase values were not available.

We used ROC curves and the AUC to assess the predictive ability of the KB approach in comparison with the actual patient outcomes. Since the KB algorithm predicts OS at various time points, we analyzed three time points―one, three and five years after diagnosis―in the whole patient cohort to test if the predictive values of the KB algorithm differed among these time points. We found that the AUC_KB_ was very similar at these time points (Supplementary Figure S1). Consequently, we chose a clinically relevant 3-year OS probability for all further analyses comparing the KB algorithm predictions with the actual outcomes.

**Comparison of the abilities to predict OS between the KB algorithm and other classifications in patients who did not die early**

We also performed the aforementioned comparisons after excluding early death patients because early death is mainly associated with advanced age, poor performance status, specific comorbidities and general health rather than the AML-specific characteristics [22,23,29]. Among 1,439 patients who did not die early, the KB approach had AUC_KB_=0.782 (95% CI, 0.758-0.806) and still outperformed both the 2017 ELN classification, with AUC_2017ELN_=0.705 (95% CI, 0.680-0.731; *p*<0.001) and the 2010 ELN classification, with AUC_2010ELN_=0.729 (95% CI, 0.693-0.745; *p*<0.001), although the differences in AUCs were less pronounced than those in the entire patient cohort.

Moreover, among younger non-early death patients, the differences in AUCs between the KB approach (AUC_KB_=0.739, 95% CI, 0.708-0.770) and the 2017 ELN classification (AUC_2017ELN_=0.696, 95% CI, 0.666-0.726, *p*=0.04) and the 2010 ELN classification (AUC_2010ELN_=0.706, 95% CI, 0.676-0.737; *p*=0.04) were even smaller. Among older patients, KB approach (AUC_KB_=0.751, 95% CI, 0.694-0.808) was still marginally better than the 2017 ELN classification (AUC_2017ELN_=0.699, 95% CI, 0.638-0.759; *p*=0.04), but it was not significantly different from the 2010 ELN classification (AUC_2010ELN_=0.721, 95% CI, 0.662-0.780; *p*=0.43).

**Additional cytogenetic and molecular markers**

The following additional karyotype subgroups, delineated in our previous study [17], that are not included in the algorithm published by Gerstung et al. [19] were also tested: atypical complex karyotype [30]; other recurrent but infrequent balanced rearrangements; unique balanced translocations and inversions; sole trisomy 4; miscellaneous sole trisomies and monosomies (not included in the KB algorithm); other, infrequent sole unbalanced abnormalities leading to gain of chromosome material; other, less frequent sole deletions or losses of chromosome material (not included in the KB algorithm); other, infrequent sole unbalanced abnormalities leading to simultaneous gain and loss of chromosome material.

We also tested mutational status of the following genes that are not included in the knowledge bank algorithm of Gerstung et al. [19]: *AKT1*, *ARAF*, *ATM*, *AXL*, *BCL2*, *BCORL1*, *BRD4*, *BRINP3*, *BTK*, *CCND1*, *CCND2*, *CSNK1A1*, *CTNNB1*, *GATA1*, *GSK3B*, *HIST1H1E*, *HNRNPK*, *IKZF3*, *IL7R*, *JAK1*, *JAK3*, *KLHL6*, *MAPK1*, *MAPK3*, *MED12*, *MYD88*, *NOTCH1*, *PIK3CD*, *PIK3CG*, *PLCG2*, *PLEKHG5*, *PRKCB*, *PRKD3*, *RAF1*, *SAMHD1*, *SETBP1*, *SMARCA2*, *SMC1A*, *SMC3*, *SYK*, *TGM7*, *TYK2*, *XPO1* and *ZMYM3*.

**Supplementary references**

1. Mayer RJ, Davis RB, Schiffer CA, Berg DT, Powell BL, Schulman P, et al. Intensive postremission chemotherapy in adults with acute myeloid leukemia. N Engl J Med. 1994;331(14):896-903.

2. Schiffer CA, Davis RB, Schulman P, Cooper B, Coyle T, Lee E, et al. Intensive post remission therapy of acute myeloid leukemia (AML) with cytoxan/etoposide (CY/VP16) and diazaquone/mitoxantrone (AZQ/MITO). Blood. 1991;78(suppl):460 (abstract 1829).

3. Moore JO, Dodge RK, Amrein PC, Kolitz J, Lee EJ, Powell B, et al. Granulocyte-colony stimulating factor (filgrastim) accelerates granulocyte recovery after intensive postremission chemotherapy for acute myeloid leukemia with aziridinyl benzoquinone and mitoxantrone: Cancer and Leukemia Group B study 9022. Blood. 1997;89(3):780-8.

4. Cassileth PA, Harrington DP, Appelbaum FR, Lazarus HM, Rowe JM, Paietta E, et al. Chemotherapy compared with autologous or allogeneic bone marrow transplantation in the management of acute myeloid leukemia in first remission. New Engl J Med. 1998;339(23):1649-56.

5. Moore JO, George SL, Dodge RK, Amrein PC, Powell BL, Kolitz JE, et al. Sequential multiagent chemotherapy is not superior to high-dose cytarabine alone as postremission intensification therapy for acute myeloid leukemia in adults under 60 years of age: Cancer and Leukemia Group B study 9222. Blood. 2005;105(9):3420-7.

6. Kolitz JE, George SL, Dodge RK, Hurd DD, Powell BL, Allen SL, et al. Dose escalation studies of cytarabine, daunorubicin, and etoposide with and without multidrug resistance modulation with PSC-833 in untreated adults with acute myeloid leukemia younger than 60 years: final induction results of Cancer and Leukemia Group B study 9621. J Clin Oncol. 2004;22(21):4290-301.

7. Walker AR, Marcucci G, Yin J, Blum W, Stock W, Kohlschmidt J, et al. Phase III randomized trial of chemotherapy with or without oblimersen in older AML patients: CALGB 10201 (Alliance). Blood Adv (In press).

8. Attar EC, Johnson JL, Amrein PC, Lozanski G, Wadleigh M, DeAngelo DJ, et al. Bortezomib added to daunorubicin and cytarabine during induction therapy and to intermediate-dose cytarabine for consolidation in patients with previously untreated acute myeloid leukemia age 60 to 75 years: CALGB (Alliance) study 10502. J Clin Oncol. 2013;31(7):923-9.

9. Blum W, Sanford BL, Klisovic R, DeAngelo DJ, Uy G, Powell BL, et al. Maintenance therapy with decitabine in younger adults with acute myeloid leukemia in first remission: A phase 2 Cancer and Leukemia Group B study (CALGB 10503). Leukemia. 2017;31(1):34-9.

10. Stone RM, Berg DT, George SL, Dodge RK, Paciucci PA, Schulman P, et al. Granulocyte-macrophage colony-stimulating factor after initial chemotherapy for elderly patients with primary acute myelogenous leukemia. N Engl J Med. 1995;332(25):1671-7.

11. Kolitz JE, George SL, Marcucci G, Vij R, Powell BL, Allen SL, et al. P-glycoprotein inhibition using valspodar (PSC-833) does not improve outcomes for patients under age 60 years with newly diagnosed acute myeloid leukemia: Cancer and Leukemia Group B study 19808. Blood. 2010;116(9):1413-21.

12. Lee EJ, George SL, Caligiuri M, Szatrowski TP, Powell BL, Lemke S, et al. Parallel phase I studies of daunorubicin given with cytarabine and etoposide with or without the multidrug resistance modulator PSC-833 in previously untreated patients 60 years of age or older with acute myeloid leukemia: results of Cancer and Leukemia Group B study 9420. J Clin Oncol. 1999;17(9):2831-9.

13. Baer MR, George SL, Caligiuri MA, Sanford BL, Bothun SM, Mrózek K, et al. Low-dose interleukin-2 immunotherapy does not improve outcome of patients age 60 years and older with acute myeloid leukemia in first complete remission: Cancer and Leukemia Group B study 9720. J Clin Oncol. 2008;26(30):4934-9.

14. Uy GL, Mandrekar SJ, Laumann K, Marcucci G, Zhao W, Levis MJ, et al. A phase 2 study incorporating sorafenib into the chemotherapy for older adults with *FLT3*-mutated acute myeloid leukemia: CALGB 11001. Blood Adv. 2017;1(5):331-40.

15. Roboz GJ, Mandrekar SJ, Desai P, Laumann K, Walker AR, Wang ES, et al. A randomized trial of 10 days of decitabine alone or with bortezomib in previously untreated older patients with acute myeloid leukemia: CALGB 11002 (Alliance). Blood Adv. 2018;2(24):3608-17.

16. Mrózek K, Carroll AJ, Maharry K, Rao KW, Patil SR, Pettenati MJ, et al. Central review of cytogenetics is necessary for cooperative group correlative and clinical studies of adult acute leukemia: the Cancer and Leukemia Group B experience. Int J Oncol. 2008;33(2):239-44.

17. Eisfeld A-K, Mrózek K, Kohlschmidt J, Nicolet D, Orwick S, Walker CJ, et al. The mutational oncoprint of recurrent cytogenetic abnormalities in adult patients with *de novo* acute myeloid*.* leukemia. Leukemia. 2017;31(10):2211-8.

18. Bill M, Nicolet D, Kohlschmidt J, Walker CJ, Mrózek K, Eisfeld A-K, et al. Mutations associated with a 17-gene leukemia stem cell score and its prognostic relevance in the context of the European LeukemiaNet classification for acute myeloid leukemia. Haematologica. 2020;105(3):721-9.

19. Gerstung M, Papaemmanuil E, Martincorena I, Bullinger L, Gaidzik VI, Paschka P, et al. Precision oncology for acute myeloid leukemia using a knowledge bank approach. Nat Genet. 2017;49(3):332-40.

20. Whitman SP, Archer KJ, Feng L, Baldus C, Becknell B, Carlson BD, et al. Absence of the wild-type allele predicts poor prognosis in adult *de novo* acute myeloid leukemia with normal cytogenetics and the internal tandem duplication of *FLT3*: a Cancer and Leukemia Group B study. Cancer Res. 2001;61(19):7233-9.

21. Marcucci G, Maharry K, Radmacher MD, Mrózek K, Vukosavljevic T, Paschka P, et al. Prognostic significance of, and gene and microRNA expression signatures associated with, CEBPA mutations in cytogenetically normal acute myeloid leukemia with high-risk molecular features: a Cancer and Leukemia Group B study. J Clin Oncol. 2008;26(31):5078-87.

22. Döhner H, Estey E, Grimwade D, Amadori S, Appelbaum FR, Büchner T, et al. Diagnosis and management of AML in adults: 2017 ELN recommendations from an international expert panel. Blood. 2017;129(4):424-47.

23. Döhner H, Estey EH, Amadori S, Appelbaum FR, Büchner T, Burnett AK, et al. Diagnosis and management of acute myeloid leukemia in adults: recommendations from an international expert panel, on behalf of the European LeukemiaNet. Blood. 2010;115(3):453-74.

24. Ng SWK, Mitchell A, Kennedy JA, Chen WC, McLeod J, Ibrahimova N, et al. A 17-gene stemness score for rapid determination of risk in acute leukaemia. Nature. 2016;540(7633):433-7.

25. Vittinghoff E, Glidden DV, Shiboski SC, McCulloch CE. Regression methods in biostatistics: linear, logistic, survival and repeated measures models. New York, NY: Springer; 2005.

26. Walter RB, Othus M, Burnett AK, Löwenberg B, Kantarjian HM, Ossenkoppele GJ, et al. Resistance prediction in AML: analysis of 4601 patients from MRC/NCRI, HOVON/SAKK, SWOG and MD Anderson Cancer Center. Leukemia. 2015;29(2):312-20.

27. Walter RB, Othus M, Borthakur G, Ravandi F, Cortes JE, Pierce SA, et al. Prediction of early death after induction therapy for newly diagnosed acute myeloid leukemia with pretreatment risk scores: a novel paradigm for treatment assignment. J Clin Oncol. 2011;29(33):4417-23.

28. Godwin CD, Othus M, Powell MA, Buckley SA, Estey EH, Walter RB. Prediction of early death in adults with relapsed or refractory acute myeloid leukemia. Leuk Lymphoma. 2016;57(10):2421-4.

29. Estey EH. Acute myeloid leukemia: 2019 update on risk-stratification and management. Am J Hematol. 2018;93(10):1267-91.

30. Mrózek K, Eisfeld A-K, Kohlschmidt J, Carroll AJ, Walker CJ, Nicolet D, et al. Complex karyotype in de novo acute myeloid leukemia: typical and atypical subtypes differ molecularly and clinically. Leukemia. 2019;33(7):1620-34.

**Supplementary Table S1.** Induction and consolidation regimes of CALGB/Alliance protocols included in this study

| **Protocol number**  **(no. of patients)** | **Treatment details** | |
| --- | --- | --- |
|  | **Induction** | **Consolidation** |
| 8525 (n=75) | AraC, 200 mg/m² per day for 7 days, continuous infusion) in combination with daunorubicin (45 mg/m² per day for 3 days) | different doses (randomized) of high dose AraC followed by maintenance treatment (3 cycles of AraC s.c.) |
| 8721 (n=2) | two courses of treatment with high dose AraC (3 g/m²) in combination with asparaginase (6,000 UI/m²) on days 2 and 9 | two to three courses of high dose AraC in combination with asparaginase |
| 8821 (n=2) | AraC (200 mg/m² per day for 7 days, continuous infusion) in combination with daunorubicin (45 mg/m² per day for 3 days) | intensive post remission therapy (randomized) with either arm 1: cyclophosphamide (50 mg/kg per day for 4 days) in combination with etoposide (2,400 mg/m²) or arm 2: diazaquone (28 mg/m² per day for 3 days) in combination with mitoxantrone (12 mg/m² per day for 3 days) |
| 8923 (n=27) | AraC (200 mg/m² per day for 5 days, continuous infusion) in combination with daunorubicin (45 mg/m² per day for 2 days) plus on day 8 - granulocyte–macrophage colony-stimulating factor (GM-CSF) | intensive post remission therapy (randomized) with either arm 1: AraC (100 mg/m² per day for 5 days, continuous infusion) or arm 2: AraC (500 mg/m² for 1 day) in combination with mitoxantrone (5 mg/m² for 1 day) |
| 9022 (n=9) | AraC (200 mg/m² per day for 5 days, continuous infusion) in combination with daunorubicin (45 mg/m² per day for 2 days) | AraC (3 g/m² per day for 3 days) in combination with etoposide (1,800 mg/m² for 1 day) and cyclophosphamide (50 mg/kg per day for 2 days). Then (randomized) arm 1: mitoxantrone (12 mg/m² per day for 3 days) in combination, diazaquone (24 mg/m² per day for 3 days) and G-CSF (5 µg/kg per day for 21 days) or arm 2: mitoxantrone (10 mg/m² per day for 3 days) in combination, diazaquone (24 mg/m² per day for 3 days) and G-CSF (5 µg/kg per day for 21 days) or arm 3: diazaquone (24 mg/m² per day for 3 days) in combination with G-CSF (5 µg/kg per day for 21 days) |
| 9120 (n=2) | AraC (100 mg/m² per day for 7 days, continuous infusion) in combination with idarubicin (12 mg/m² per day for 3 days) | AraC (100 mg/m² per day for 7 days, continuous infusion) in combination with idarubicin (12 mg/m² per day for 3 days) followed by autologous or allogeneic bone marrow transplantation or AraC (3 g/m² per day for 6 days) |
| 9222 (n=84) | AraC (200 mg/m² per day for 7 days, continuous infusion) in combination with daunorubicin (45 mg/m² per day for 3 days) | different doses (randomized) of high dose AraC consisting of either arm 1: 2 cycles of AraC (3 g/m² twice per day for 3 days) or arm 2: etoposide (1,800 mg/m² for 1 day, continuous infusion) in combination with cyclophosphamide (50 mg/kg per day for 2 days) |
| 9420 (n=22) | AraC (100 mg/m² per day for 7 days, continuous infusion) in combination with daunorubicin (45 mg/m² per day for 3 days) and etoposide (100 mg/m² for 3 days) | AraC (100 mg/m² per day for 7 days, continuous infusion) in combination with daunorubicin (45 mg/m² per day for 3 days) and etoposide (100 mg/m² for 3 days) with or without (randomized) the multidrug resistance protein modulator PSC-833 followed by low dose of rIL-2 immunotherapy |
| 9621 (n=172) | AraC (100 mg/m² per day for 7 days, continuous infusion) in combination with daunorubicin (60 mg/m² per day for 3 days) and etoposide (100 mg/m² for 3 days) with or without PSC-833 (valspodar) | Patients with CBF-AML received three cycles of AraC (3 g/m^2^ over 3 h, every 12 h, on days 1, 3 and 5). All other patients were assigned to receive AraC (2 g/m² twice per day for 4 days) in combination with etoposide (40 mg/kg for 4 days, continuous infusion) followed by autologous stem cell transplantation |
| 9720 (n=233) | AraC (100 mg/m² per day for 7 days, continuous infusion) in combination with daunorubicin (60 mg/m² per day for 3 days) and etoposide (100 mg/m² for 3 days) | AraC (100 mg/m² per day for 7 days, continuous infusion) in combination with daunorubicin (45 mg/m² per day for 3 days) and etoposide (100 mg/m² for 3 days) with or without (randomized) the multidrug resistance protein modulator PSC-833 followed by low dose of rIL-2 immunotherapy |
| 10201 (n=166) | AraC (100 mg/m² per day for 7 days, continuous infusion) in combination with daunorubicin (60 mg/m² per day for 3 days) with or without (randomized) G3139, a BCL2 antisense oligodeoxynucleotide | AraC (2,000 mg/m² per day for 5 days) with or without (randomized) G3139, a BCL2 antisense oligodeoxynucleotide |
| 10502 (n=38) | AraC (100 mg/m² per day for 7 days, continuous infusion) in combination with daunorubicin (60 mg/m² per day for 3 days) and bortezomib (1.3 g/m² for 4 days) | AraC (2,000 mg/m² per day for 5 days) in combination with bortezomib (1.3 g/m² for 4 days) |
| 10503 (n=312) | AraC (100 mg/m² per day for 7 days, continuous infusion) in combination with daunorubicin (90 mg/m² per day for 3 days) and etoposide (100 mg/m² for 3 days) | Patients with CBF-AML, received three cycles AraC (3 g/m^2^ over 3 h, every 12 h, on days 1, 3 and 5). All other patients underwent chemo-mobilization with AraC (2 g/m² twice per day for 4 days) in combination with etoposide (40 mg/kg for 4 days, continuous infusion) followed by autologous stem cell transplantation |
| 10603 (n=71) | AraC (200 mg/m² per day for 7 days, continuous infusion) in combination with daunorubicin (60 mg/m² per day for 3 days) with or without (randomized) midostaurin (50 mg twice per day for 14 days) | Four cycles: AraC (3 g/m² per day for 3 days) with or without (randomized) midostaurin (50 mg twice per day for 14 days |
| 10801 (n=1) | AraC (200 mg/m² per day for 7 days, continuous infusion) in combination with daunorubicin (60 mg/m² per day for 3 days) and dasatinib (100 mg per day for 14 days) | Four cycles: AraC (3 g/m² per day for 3 days) in combination with dasatinib (100 mg per day for 14 days) |
| 11001 (n=10) | AraC (100 mg/m² per day for 7 days, continuous infusion) in combination with daunorubicin (60 mg/m² per day for 3 days) with or without (randomized) sorafenib (400 mg twice per day for 7 days) | Two cycles: AraC (2 g/m² per day for 3 days) in combination with sorafenib (400 mg twice per day for 7 days) |
| 11002 (n=38) | decitabine (20 mg/m² per day for 10 days) with or without (randomized) bortezomib (1.3 g/m² for 4 days) | decitabine (20 mg/m² per day for 10 days) with or without (randomized) bortezomib (1.3 g/m² for 4 days) |
| 19808 (n=140) | AraC (100 mg/m² per day for 7 days, continuous infusion) in combination with daunorubicin (90 mg/m² per day for 3 days) and etoposide (100 mg/m² for 3 days) with or without PSC-833 (valspodar) | Patients with CBF-AML received three cycles of AraC (3 g/m^2^ over 3 h, every 12 h, on days 1, 3 and 5). Patients with non-CBF-AML received AraC (2 g/m² twice per day for 4 days) in combination with etoposide (40 mg/kg for 4 days, continuous infusion) followed by autologous stem cell transplantation |

AraC, cytarabine; CBF-AML, core-binding factor (CBF) AML.

**Supplementary Table S2.** Pretreatment clinical characteristics of AML patients included in our analyses

| **Characteristic** | **All patients**  ***n*=1612** | **Younger patients**  ***n*=1043** | **Older patients**  ***n*=569** |
| --- | --- | --- | --- |
| Age, years |  |  |  |
| Median  Range | 53  17-92 | 45  17-59 | 69  60-92 |
| Sex, no. (%) |  |  |  |
| Male | 899 (56) | 569 (55) | 330 (58) |
| Female | 713 (44) | 474 (45) | 239 (42) |
| Hemoglobin, g/dl |  |  |  |
| Median   Range | 9.2  2.3-25.1 | 9.2  2.3-25.1 | 9.3  3.0-15.0 |
| Platelet count, x10^9^/l |  |  |  |
| Median   Range | 54  4-989 | 53  4-648 | 59  4-989 |
| WBC count, x10^9^/l |  |  |  |
| Median   Range | 23.5  0.4-560.0 | 24.0  0.4-560.0 | 20.2  0.4-450.0 |
| Blood blasts, % |  |  |  |
| Median   Range | 52  0-99 | 55  0-99 | 41  0-99 |
| Bone marrow blasts, % |  |  |  |
| Median   Range | 66  0-99 | 67  0-99 | 64  0-99 |
| ELN 2017 risk group, %  Favorable  Intermediate  Adverse | 41  37  36 | 47  23  30 | 30  25  45 |
| ELN 2010 risk group, %  Favorable  Intermediate-I  Intermediate-II  Adverse | 34  22  21  23 | 40  20  19  21 | 22  25  25  27 |
| Extramedullary involvement, no. (%) | 25 | 27 | 22 |

ELN, European LeukemiaNet; WBC, white blood cell.

**Supplementary Table S3.** Treatment outcomes of AML patients included in our analyses

| **Endpoint** | **All patients**  ***n*=1612** | **Younger patients**  ***n*=1043** | **Older patients**  ***n*=569** |
| --- | --- | --- | --- |
| Complete remission, no. (%) | 1022 (63) | 767 (73) | 258 (45) |
| Disease-free survival   Median, years  % disease-free at 1 year (95% CI)  % disease-free at 3 years (95% CI)  % disease-free at 5 years (95% CI) | 1.1  53 (50-56)  34 (31-37)  31 (28-34) | 1.5   58 (55-62)   40 (37-43)   37 (33-40) | 0.6   37 (31-43)   15 (11-20)   13 (9-17) |
| Overall survival   Median, years  % alive at 1 year (95% CI)  % alive at 3 years (95% CI)  % alive at 5 years (95% CI) | 1.2  55 (52-57)  32 (30-35)  28 (26-30) | 2.0   66 (63-69)   43 (40-46)   38 (35-41) | 0.6   33 (29-37)   13 (10-16)   9 (7-11) |

CI, confidence interval.

**Supplementary Table S4.** Characteristics that can be provided to the knowledge bank algorithm*

| Demographic data | | | | | | | | |
| --- | --- | --- | --- | --- | --- | --- | --- | --- |
| **Age at diagnosis** | | | | | **Sex** | | | |
| Clinical data | | | | | | | | |
| **ECOG performance status** | | | **WBC** | | | **Platelet count** | | |
| **Bone marrow blasts** | | | Lactic acid dehydrogenase | | | **Splenomegaly** | | |
| **Blood blasts** | | | **Hemoglobin** | | | **AML type**^†^ | | |
| Cytogenetics | | | | | | | | |
| **inv(3)/t(3;3)** | | **t(v;11)** | | | **-12/12p-/abn(12p)** | | **-Y** | |
| **t(9;22)** | | **-5/5q-** | | | **+13** | | **abn(3q) (other)** | |
| **t(15;17)** | | **-7** | | | **-17/17p-/abn(17p)** | | **+11/11q+** | |
| **t(8;21)** | | **7q-** | | | **-18/18q-** | | **-4/4q-/abn(4q)** | |
| **inv(16)/t(16;16)** | | **abn(7) (other)** | | | **-20/20q-** | | **complex karyotype** | |
| **t(6;9)** | | **+8/8q+** | | | **+21** | |  | |
| **t(9;11)** | | **9q-** | | | **+22** | |  | |
| Molecular genetics^‡^ | | | | | | | | |
| ***ASXL1*** | ***EZH2*** | | | *MLL2* | | ***RAD21*** | | ***U2AF2*** |
| *ATRX* | ***FBXW7*** | | | *MLL3* | | *RB1* | | ***WT1*** |
| ***BCOR*** | ***GATA2*** | | | *MLL5* | | ***RUNX1*** | | ***ZRSR2*** |
| ***BRAF*** | *GNAS* | | | ***MPL*** | | ***SF1*** | | ***CEBPA* (mono)** |
| ***CBL*** | ***IDH1*** | | | ***MYC*** | | ***SF3A1*** | | ***CEBPA* (bi-allelic)** |
| *CBLB* | ***IKZF1*** | | | ***NF1*** | | ***SF3B1*** | | ***FLT3* (ITD)** |
| *CDKN2A* | ***JAK2*** | | | ***NPM1*** | | ***SRSF2*** | | ***FLT3* (TKD)** |
| *CREBBP* | *KDM5A* | | | ***NRAS*** | | ***SH2B3*** | | ***FLT3* (other)** |
| *CUX1* | *KDM6A* | | | ***PHF6*** | | ***STAG2*** | | ***IDH2* (p172)** |
| ***DNMT3A*** | ***KIT*** | | | *PRPF40B* | | ***TET2*** | | ***IDH2* (p140)** |
| *EP300* | ***KRAS*** | | | ***PTEN*** | | ***TP53*** | |  |
| ***ETV6*** | ***MLL*** | | | ***PTPN11*** | | ***U2AF1*** | |  |

* Characteristics available for the majority of patients included in our study are set in bold

type font.

^†^ *De novo*, secondary or therapy-related AML.

^‡^ Underlined are the g enes whose mutations had the highest predictive value in the KB

algorithm published by Gerstung et al. [19].

**Supplementary Figure S1.** The receiver operating characteristic (ROC) curves illustrating the ability of the KB algorithm to predict overall survival rates in the whole cohort of adult patients with AML **a** at one year [AUC_KB_=0.781, 95% confidence interval (CI), 0.759-0.803], **b** at three years (AUC_KB_=0.779, 95% CI, 0.777-0.821) and **c** at five years (AUC_KB_=0.806, 95% CI, 0.783-0.828).


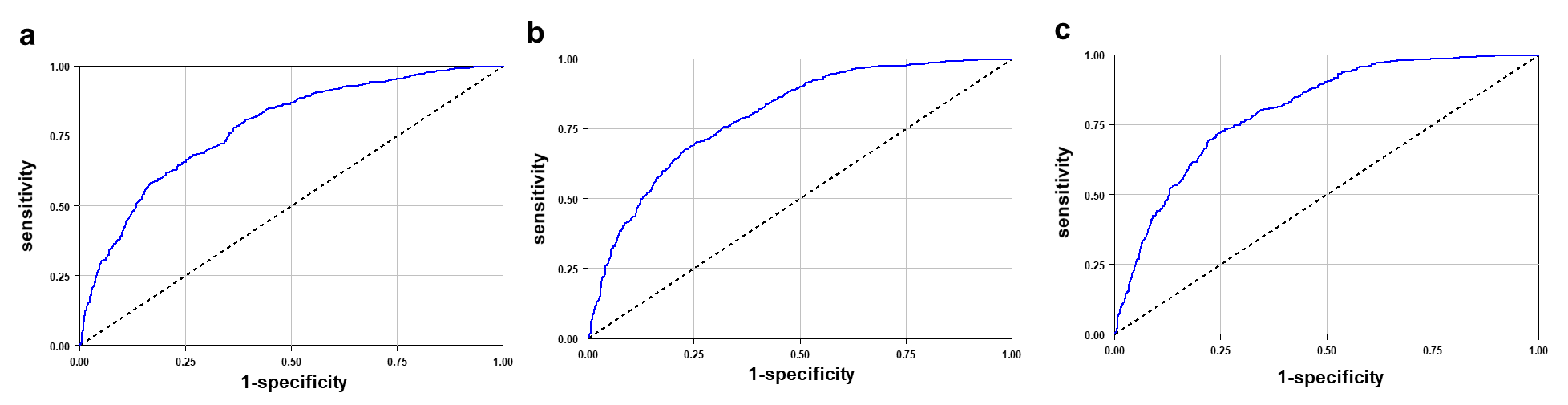

Supplement: Supplementary file 1 — Additional file 1. Supplementary Material. [file 13045_2021_1118_MOESM1_ESM.docx]
